# Castor Oil Injection Vomited

**Published:** 1874-11

**Authors:** H. M. Hearn


Castor Oil Vomited up Forty Minutes after being Injected into the Rectum.—*By H. M. Hearn, M.D.*—On the 30th of July, 1874, I was called in great haste to Mrs. E. H., aged 47, who was complaining of great nausea and incessant retching—vomiting a glairy mucus, and also everything that was taken into the stomach; hence we were forced to resort to the use of injections, both hypodermical and rectal.

There being considerable constipation, it was evidently necessary to open the bowels as speedily as possible. For this purpose we used the ordinary injection—castor oil forming a part, and which was vomited up within forty minutes afterwards. The attention of Dr. Fletcher (who was present in consultation) was immediately called to the appearance of the ejected matter in the night-glass, when he carefully separated the oil from the mucus, and bottled near a tablespoonful, which he took to his office and thoroughly examined. It was, beyond all doubt, the oil which had been injected, as she had taken no oil into the stomach.

Her bowels were freely purged shortly afterwards.

She has been a very healthy woman until within the last twelve months, during which time she has been suffering some irregularity in her menstrual periods.—*Nashville Journal of Medicine and Surgery.*

A Hint in Giving Iodide of Potassium.—Sir James Paget was the first to call the attention of the medical profession to the following interesting fact—namely, that carbonate of ammonia greatly increases the therapeutic action of iodide of potassium. I have had extensive experience in the treatment of syphilis, and have tried it with the best results, and find that five grains of iodide of potassium, combined with three grains of carbonate of ammonia, are equal to eight grains of the potassium salt administered in the ordinary way.—*Cincinnati Lancet and Observer.*

